# Early detection of nerve involvement in presymptomatic *TTR* mutation carriers: exploring potential markers of disease onset

**DOI:** 10.1007/s10072-023-07177-x

**Published:** 2023-11-08

**Authors:** Angela Romano, Valeria Guglielmino, Giulia Bisogni, Andrea Di Paolantonio, Andrea Truini, Angelo Maria Minnella, Maria Ausilia Sciarrone, Francesca Vitali, Martina Maceroni, Eleonora Galosi, Mario Sabatelli, Marco Luigetti

**Affiliations:** 1 UOC Neurologia, Fondazione Policlinico Universitario Agostino Gemelli IRCCS, Largo Agostino Gemelli, 8, 00168 Rome, Italy; 2 Dipartimento Di Neuroscienze, Università Cattolica del Sacro Cuore, Rome, Italy; 3 Centro Clinico NeMO Adulti, Fondazione Serena Onlus-Fondazione Policlinico Universitario Agostino Gemelli IRCCS, Rome, Italy; 4 UO Neurologia, Fondazione Poliambulanza, Brescia, Italy; 5 Department of Human Neuroscience, Sapienza University, Rome, Italy; 6 UOC Oftalmologia, Fondazione Policlinico Universitario Agostino Gemelli IRCCS, Rome, Italy

**Keywords:** Hereditary transthyretin amyloidosis, ATTRv-PN, Presymptomatic carriers, Disease onset biomarkers, Early diagnosis

## Abstract

**Background:**

Hereditary transthyretin (ATTRv) amyloidosis is a heterogeneous, progressive, multisystemic disease with a life-threatening course if left untreated. Given the current availability of effective therapies, close follow-up of presymptomatic *TTR* mutation carriers is essential to recognize disease onset at the earliest sign. In addition to routine techniques, in recent years several novel tools have been proposed, although a consensus on their use has not been reached yet. In this paper, we aimed to evaluate possible markers of neuropathic disease onset intended to discriminate clinically asymptomatic carriers from early symptomatic patients, thus allowing timely treatment initiation.

**Methods:**

Thirty-eight presymptomatic carriers were enrolled. Clinical and electrophysiological findings at first evaluation and follow-up were collected. All carriers underwent an extensive clinical and instrumental evaluation according to the standard clinical practice. One or more non-routine investigations, whose use in this field is not yet validated (henceforth “unconventional”), were additionally assessed in a subgroup of individuals.

**Results:**

Based on the exclusive use of routine investigations, it was possible to define disease onset in 4/38 carriers during the follow-up. Employing additionally one or more “unconventional” tests, abnormal findings, indicative of a possible “conversion” to symptomatic disease, were detected in further 12 cases. More than half of our study cohort showed findings suggestive of small nerve fiber (SF) involvement at either invasive or non-invasive tests.

**Conclusions:**

A close, multidisciplinary monitoring of presymptomatic *TTR* mutation carriers is fundamental, and diagnostic workup should include both routine and “unconventional” tests. Assessment of SF involvement is important also in non-endemic countries.

**Supplementary Information:**

The online version contains supplementary material available at 10.1007/s10072-023-07177-x.

## Introduction

*Hereditary transthyretin* (*ATTRv*, *v* for “variant”) *amyloidosis* is a clinically and genetically heterogeneous, adult-onset, autosomal-dominant disease with variable penetrance, caused by mutations in the gene encoding transthyretin (*TTR*). This severe disease results from the multisystemic extracellular deposition of misfolded variant TTR as insoluble amyloid fibrils, causing progressive, irreversible organ dysfunction, with a poor prognosis if untreated [1–3].

Nowadays, the advent of new drugs acting at distinct stages of the amyloid cascade is dramatically changing disease course and clinical outcome [4].

This has raised the need for reliable disease biomarkers aimed at monitoring disease progression and objectively evaluating the response to pharmacological treatment. Similarly, the availability of effective therapies has pointed out the importance of the genetic counseling of at-risk relatives of ATTRv patients and the regular monitoring of identified carriers of pathogenic *TTR* variants [5]. Since the clinical benefit of all disease-modifying treatments (DMTs) is expected to be higher the sooner they are started, strict monitoring of presymptomatic carriers, especially of those close to the predicted age of disease onset (PADO), is indeed essential to maximize the effectiveness of treatment.

In 2018, a group of experts met to reach a consensus on minimum criteria for defining disease onset and the best approach for the targeted monitoring of presymptomatic carriers [6]. Such recommendations suggest annual follow-up of presymptomatic *TTR* mutation carriers starting 10 years before the PADO, suggesting a list of investigations useful for this purpose. Given the great clinical variability of the disease, a multidisciplinary approach is mandatory, and the type of tests to be used should be tailored according to the expected phenotypic presentation for that specific genotype in each geographic region. The definition of “disease onset” is based on a combination of signs/symptoms and abnormal test findings [6].

However, to date, there are no reliable biomarkers able to identify the conversion from an asymptomatic status to an overt illness.

In the last few years, in addition to the recommended standard tests, further exams have emerged and have been gaining attention as potential biomarkers of both disease onset and progression, such as skin biopsy [7, 8], quantitative sensory testing (QST) [9, 10], nerve ultrasound [11, 12], magnetic resonance (MRI) neurography [13], muscle MRI [14, 15], serum or plasma levels of neurofilament light chain (NfL) [16–20], and extensive ophthalmological evaluation [21].

In the present paper, we aimed to evaluate the potential usefulness and reliability of some still unvalidated (henceforth, “*unconventional*”) investigations, in addition to the standard tests proposed by the 2018 Consensus, in the early diagnosis of symptomatic ATTRv amyloidosis–related polyneuropathy (ATTRv-PN) in order to permit timely treatment initiation.

## Patients and methods

### Study design and population

We carried out a single-center, observational, longitudinal, prospective study on a group of presymptomatic *TTR* mutation carriers identified in the context of familiar genetic screening. Our primary aim was to evaluate the percentage of carriers who converted to symptomatic ATTRv-PN during the follow-up according to Consensus Criteria and recommended routine tests. Moreover, we tried to assess the possible application of some still unvalidated tests in a subgroup of carriers with normal or no definitive findings on traditional investigations. This was done to evaluate if some of these “unconventional” tests could be more reliable for an early diagnosis, thus expanding the proposed diagnostic tools and prompting earlier initiation of DMTs.

The study was conducted at the Institute of Neurology of the “Fondazione Policlinico Universitario Agostino Gemelli IRCCS” in Rome, Italy. The local Ethics Committee approved the study (Prot. ID 4108), and all participants signed a written informed consent. All procedures were conducted in compliance with the ethical standards of the Declaration of Helsinki.

For the present study, we consecutively recruited subjects aged > 18 years with a confirmed pathogenic *TTR* variant and neither signs nor symptoms of multisystemic involvement *definitely* related to ATTRv amyloidosis at baseline evaluation, in regular follow-up at our Institute.

For each subject, the following data were collected: *TTR* variant, sex, age both at baseline evaluation and at the time of conversion (if applicable) or last follow-up, age at onset in other relatives, and the sex of the transmitting parent where available. PADO was estimated according to the literature [6], using the age of onset of the index case (or the youngest affected relative if many).

We recorded any concomitant known cause or risk factor for neuropathy, such as diabetes, paraproteinemia, alcohol abuse, vitamin B12 or B9 deficiencies, thyroid or chronic kidney diseases, autoimmune disorders, or chronic viral infections (including HCV and HIV).

All enrolled subjects were evaluated roughly yearly through an extensive clinical and instrumental neurological evaluation according to the standard clinical practice [6]. A subgroup of individuals with normal or no definitive findings on routine tests was also evaluated using one or more ancillary tests aimed at exploring large or small nerve fiber involvement.

Presymptomatic carriers, especially those with a *TTR* variant usually associated with a mixed or cardiac phenotype, were also referred to a cardiologist with expertise in amyloid cardiomyopathies for baseline assessment and follow-up. However, for the present paper, we chose to focus only on the polyneuropathy (PN) assessment.

### Clinical evaluation and conventional investigations

All subjects underwent a complete general and neurological examination (including the assessment of the Neuropathy Impairment Score, or NIS) [22]. Any change in blood pressure within 3 min of standing from a supine position was recorded to look for orthostatic hypotension [23].

Clinical questionnaires, commonly used to assess the quality of life and detect sensorimotor and/or autonomic symptoms in ATTRv amyloidosis, were administered to all participants. These included the Norfolk Quality of Life-Diabetic Neuropathy (QOL-DN), Questionnaire Douleur neuropathique 4 (DN4), and the Compound Autonomic Dysfunction Test (CADT) [24–26].

Conventional nerve conduction studies (NCSs) were performed employing a Dantec™ Keypoint® EMG/NCS equipment. NCSs of bilateral sural and tibial nerves and unilateral (*non-dominant hand*) sensory and motor ulnar and median nerves were done according to standard protocols, using surface stimulating and recording electrodes [27]. The sural sensory nerve action potential (SNAP) was recorded antidromically, whereas upper limb’ SNAPs were recorded using orthodromic techniques. Age- and sex-adjusted normative values of our laboratory were used [28, 29].

The sudomotor function was assessed by Sudoscan™ (Impeto Medical, Paris, France), a non-invasive, rapid test measuring electrochemical skin conductance (ESC) of feet and hands in response to a low voltage electrical stimulus. Mean ESC values for the feet and the hands were recorded. For interpreting the results, we referred to the manufacturer’s reference intervals, obtained on a large adult population [30]. We considered *definitely* abnormal those values in the “red/lower” range (feet ESC≤50 μS, hands ESC≤40 μS, suggestive of severe sudomotor dysfunction). Conversely, for values in the “yellow/intermediate” range (feet ESC 51–69 μS, hands ESC 41–59 μS, indicative of mild/borderline sudomotor dysfunction), we considered these findings as suggestive of an early small nerve fiber involvement only if at least another additional test was abnormal as well.

### “Unconventional tests” evaluating large nerve fibers

#### Dorsal sural nerve (DSN)

NCSs of the DSN were performed in a subgroup of enrolled subjects having normal findings on conventional NCSs. Bilateral DSN was recorded antidromically, according to standard protocols [31].

Age- and sex-related normative values of our laboratory were used. The ratio between the SNAP amplitudes of the sural nerve and the DSN was also calculated, using a cut-off value of 4.17 [31]. We considered definitely pathological only those cases where both the age-/sex-adjusted DSN SNAP and the sural/DSN SNAP ratio were abnormal.

### “Unconventional tests” evaluating small nerve fibers

#### Cutaneous silent period (CSP)

The CSP was obtained by stimulating the third and fourth digits with ring electrodes and recording from the abductor brevis pollicis (ABP) and the abductor digiti minimi (ADM) muscles of the non-dominant hand, respectively, using surface electrodes, in compliance with standard protocols [32, 33]. The *onset latency* was measured at the beginning of the abrupt EMG suppression, and the *end latency* at the recovery of EMG activity in each trace. The arithmetic difference between the 2 latencies defined *CSP duration*.

#### Skin biopsy

Skin biopsy was performed and processed at Sapienza University in a subgroup of carriers. Skin specimens were obtained using a sterile 3-mm punch from a distal (*calf*) and a proximal (*thigh*) site, and processed according to protocols described elsewhere [7].

Intraepidermal nerve fiber density (IENFD) was measured as the number of fibers/mm. Age-/sex-adjusted international accepted normative values for both distal and proximal IENFD were used [34]. The ratio between distal and proximal IENFD (*leg/thigh ratio*) was also calculated to evaluate whether small nerve fiber loss was consistent with a length-dependent pattern (cut-off value 0.48) [34].

#### In vivo* corneal confocal microscopy (CCM)*

CCM was performed in a subgroup of carriers with the Heidelberg Retinal Tomograph Rostock Corneal Module (HRT-RCM) confocal laser scanning microscope (Heidelberg Engineering, GmBH, Dossenheim, Germany) [21]. A qualitative evaluation was performed by an expert ophthalmologist who analyzed the subepithelial nervous plexus (extension and density), the morphology of corneal nerves (looking for nerve segmentation and/or fragmentation), and the corneal nerve branch density.

#### Serum levels of NfL (sNfL)

Serum aliquots for determination of NfL levels were available for a subgroup of carriers.

Blood samples were collected into serum separator tubes, clot for 30 min at room temperature, and then centrifuged at 2500 rpm for 15 min. Serum aliquots were stored at − 80 °C until analysis. Serum NfL concentration was measured using the Simple Plex™ cartridge-based Assay on Ella™ platform (ProteinSimple, San Jose, CA, USA), according to the manufacturer’s instructions.

A sNfL cut-off of 37.10 pg/mL was used, as recently published [35].

### Statistical analysis

The sample was described in its clinical and demographic characteristics using descriptive statistics techniques. Qualitative variables were described with absolute frequencies and percentages (%). Quantitative variables were summarized as mean ± standard deviation (SD), and/or as median and interquartile range [IQR].

For categorical variables, comparisons were performed by applying the chi-square test, or the Fisher exact test, as appropriate. For quantitative variables, the comparison of means between two independent groups was performed with the independent samples *t*-test or the Mann–Whitney test, as appropriate. The normal distribution of quantitative data and homogeneity of variance were assessed with the Shapiro–Wilk test and Levene’s test, respectively.

Statistical analyses were performed using IBM® SPSS® Statistics version 25.0. The significance level was set at *p* < 0.05.

## Results

### Study population

Thirty-eight presymptomatic carriers were enrolled. Twenty of them (52.60%) were male, *vs.* eighteen females (47.40%), with a male-to-female ratio of 1.11. All the enrolled carriers were Caucasian (Italian), and all of them harbored a late-onset *TTR* variant, except for one female (F#21) carrying a V30M variant and having a PADO of 35 years. V30M and F64L were the most common *TTR* variants, being identified in 21 (55.3%) and 12 (31.6%) individuals, respectively.

The demographic characteristics of the study cohort are summarized in Table 1.

**Table 1:** Demographic data of our study cohort. Qualitative variables are described with absolute frequencies and percentages (%). Quantitative variables are summarized as mean ± standard deviation; median [*IQR*, interquartile range]

	Presymptomatic *TTR* mutation carriers (*n* = 38)
Male sex	20 (52.60%)
*TTR* genotype	
- Val30Met	21 (55.3%)
- Phe64Leu	12 (31.6%)
- Glu89Gln	2 (5.3%)
- Ala120Ser	1 (2.6%)
- Val122Ile	1 (2.6%)
- Ile68Leu	1 (2.6%)
Inheritance	
- Paternal line	19 (50.0%)
- Maternal line	11 (28.9%)
- Unknown^a^	8 (21.1%)
Age at PST (*years*)	51.21 ± 12.85; 49.00 [40.00–63.00]
Age at first evaluation (*years*)	52.61 ± 12.14; 50.00 [42.75–63.25]
PADO (*years*)	60.00 ± 8.50; 60.50 [54.00–63.50]
Time-to-PADO (*years*)	− 5.26 ± 11.90; − 7.00 [− 14.00 to 5.75]
Follow-up (*months*)	29.66 ± 30.67; 17.00 [3.75–55.00]

^a^This subgroup of carriers had undergone a PST as siblings of an affected patient, without a known or confirmed diagnosis in their ancestors

Abbreviations: *TTR*, transthyretin; *PST*, presymptomatic genetic test; *PADO*, predicted age of disease onset

### Routine clinical and instrumental evaluation

Enrolled carriers were evaluated yearly for a mean follow-up of 29.7 (± 30.7) months.

During the monitoring, 14/38 carriers (36.8%) complained of symptoms *possibly* related to ATTRv (mainly distal limb dysesthesia and/or paresthesia, except for one case with gastrointestinal symptoms).

Clinical examination and Sudoscan at baseline were unremarkable in all enrolled subjects. Conventional NCSs at t0 excluded a polyneuropathy in the whole cohort.

During the follow-up, 2 siblings carrying a F64L variant [one female (F#13) and one male (M#15), with a paternal inheritance and a PADO of 76 years] developed length-dependent sensory polyneuropathy as the first disease manifestation, at 58 and 51 years old, respectively (Supplementary Table 1), and they could start a DMT according to minimum Consensus Criteria.

Conversely, in the monitoring, Sudoscan showed abnormal findings (either in the “red/lower” or “yellow/intermediate” range) in 13/38 carriers (34.21%, Table 2 and Supplementary Table 1), most commonly after their PADO (10/13 *vs* 3/13 before PADO, *p* = 0.001).

**Table 2:** Tests employed to assess large and small nerve fibers involvement in our study cohort, and count/percentage of abnormal findings. Both routine and “unconventional” tests (*in italics*) are reported

	Examined cases [count (% in relation to the whole study cohort)]	Abnormal findings [count (% in relation to the examined cases)]
*Investigations aimed at exploring large nerve fibers*		
Routine NCSs	38 (100%)	2 (5.26%)
*NCSs of DSN*	30 (78.95%)	10 (33.33%)^a^
*Investigations aimed at exploring small nerve fibers*		
Sudoscan	38 (100%)	13 (34.21%)^b^
*CSP*	30 (78.95%)	2 (6.67%)^c^
*Skin biopsy*	19 (50.0%)	11 (57.89%)
*CCM*	7 (18.42%)	7 (100%)

^a^Only cases where both the age-/sex-adjusted DSN SNAP and the sural/DSN SNAP ratio were abnormal were considered definitely pathological

^b^Both cases indicative of severe and mild/borderline sudomotor dysfunction are included

^c^Given the absence of normative data for CSP, we considered definitely abnormal only those cases for which it was absent

Abbreviations: *NCSs*, nerve conduction studies; *DSN*, dorsal sural nerve; *CSP*, cutaneous silent period; *CCM*, in vivo corneal confocal microscopy

Namely, two carriers (F#28, and M#20), both carrying a V30M variant (a 71-year-old woman with a familiar age at onset of 63 years in the oldest affected brother and a 54-year-old man with a paternal inheritance and a PADO of 60 years), showed definitely abnormal findings on Sudoscan (i.e., in the “red/lower” range, suggestive of severe sudomotor dysfunction) as the first sign of neurological impairment; conventional NCSs were normal in both.

Additionally, Sudoscan revealed borderline/mild abnormal ESC values (i.e., in the “yellow/intermediate” range) at lower and/or upper limbs in further 11 cases (*feet ESC*: mean 63.55 ± 7.38 μS; *hands ESC*: mean 61.09 ± 10.71 μS). Among this latter group, 8 agreed to undergo skin biopsy, which confirmed abnormal findings in 6 of them. Overall, 7/11 individuals with “intermediate” ESC values had at least another abnormal test (namely, skin biopsy in 6 cases, NCSs of DSN in 3 cases, CCM in 2 cases). As concerns the remaining 4 carriers (F#3, M#4, M#8, M#27), showing normal findings on all other neurological investigations besides Sudoscan, none of them complained of any symptoms, and none of them was then considered “converted” to symptomatic disease.

Interestingly, in 2/11 cases (M#26, M#27), abnormal Sudoscan findings were suggestive of a non-length-dependent pattern, but both carriers refused to undergo skin biopsy.

#### Overall conventional diagnostic tests

Globally, considering routine tests and minimum Consensus Criteria, 4 out of 38 subjects (10.53%) had a confirmed diagnosis of symptomatic ATTRv-PN and could start specific anti-amyloid treatment.

Among these, 2 patients initially developed length-dependent sensory PN, whereas the remaining 2 patients showed small nerve fiber involvement as the first sign of neurological impairment.

The mean age at conversion in this subgroup was 58.50 ± 8.81 years (median 56.00, IQR 51.75–67.75). The mean PADO was 68.75 ± 8.46 years (median 69.50, IQR 60.75–76.00), with a mean time-to-PADO of − 10.25 ± 14.48 years (median − 12.00. IQR − 23.25 to 4.50). In 3 out of 4 cases (75%), conversion to symptomatic disease was detected before the individual’s PADO (up to 25 years before).

### “Unconventional” tests

#### DSN

Evaluation of DSN was available in 30/38 enrolled subjects, all of which with normal conventional NCSs. Ten of the examined subjects (33.33%) had both abnormal “corrected” SNAP amplitude and sural/DSN SNAP ratio (mean ratio 4.57 ± 1.47, median 4.68, IQR 3.14–5.30).

### CSP

CSP was obtained in 30 carriers, and it was absent from both ABP and ADM in 2 of them (Table 2 and Supplementary Table 1). Both cases showed abnormal findings on other tests as well.

Namely, skin biopsy proved signs of skin denervation in one case (a 60-year-old man carrying a Val122Ile variant, with a PADO of 72 years, complaining of distal limb paresthesia; M#35). Additionally, he showed abnormal NCSs of DSN [36].

The other carrier (a 45-year-old man harboring a Glu89Gln variant, with a PADO of 56 years; M#34) complained of severe gastrointestinal symptoms (namely daily diarrhea), suggesting a possible autonomic dysfunction. The hypothesis of a prevalent small nerve fiber involvement was further supported by the evidence of abnormal findings on CCM. A skin biopsy has been then recommended but the patient refused.

As concerns the remaining 28 carriers, given the absence of normative values, we cannot draw any conclusion. However, they showed a higher onset latency and a tendency towards a shorter duration as compared to a control group, as already published [37].

#### Skin biopsy

Skin biopsy was performed in 19/38 subjects (Table 2 and Supplementary Table 1). The mean age at skin biopsy was 54.74 ± 12.81 years. Considering age- and sex-adjusted normative values, a reduced IEFND was evident in 11/19 (57.89%), in 5 of whom before their PADO (*vs* 6 after PADO, *p* = 0.690). Actually, a 42-year-old man carrying a Phe64Leu variant (M#16) showed abnormal findings at a time-to-PADO of approximately − 20 years.

The mean proximal (thigh) IEFND was 10.23 ± 2.07 fibers/mm (median 10.62, IQR 9.34–11.74). The mean distal (leg) IEFND was 7.14 ± 1.55 fibers/mm (median 7.12, IQR 6.37–8.33). The mean leg/thigh IEFND ratio was 0.71 ± 0.24 fibers/mm (median 0.68, IQR 0.56–0.86). Notably, in 9/11 carriers, skin denervation was not consistent with a length-dependent pattern (leg/thigh ratio > 0.48).

#### CCM

Fourteen eyes of 7 carriers were analyzed using CCM. None of them complained of ocular symptoms. All the examined eyes (100%) presented a rarefied subepithelial nervous plexus (for extension and density), nerve segmentation and/or fragmentation (increased beads), and a reduced branch density (Table 2 and Supplementary Table 1).

Six out of 7 carriers underwent skin biopsy as well, proving abnormal in 3 of them (50.0%).

#### Overall “unconventional” diagnostic tests

Globally, considering “unconventional” diagnostic tests in addition to conventional exams, 16 individuals (42.10%) could have been considered affected, as compared to 4 individuals (10.53%) considered “converted” based on only routine tests proposed by the Consensus. Thus, a diagnosis of disease onset could have been reached in further 12 cases [in 5 before their PADO (up to 21 years before) *vs* 7 after PADO, *p* = 0.163].

The mean age at conversion in this further subgroup of 12 individuals was 62.08 ± 12.49 years (median 65.50, IQR 48.50–72.75). The mean PADO was 61.42 ± 6.36 years (median 62.00, IQR 55.25–66.00), with a mean time-to-PADO of 0.67 ± 11.52 years (median 4.50. IQR − 10.25 to 11.00).

### Assessment of large and small nerve fiber involvement

Overall, large nerve fiber involvement was evident in 12/38 carriers (31.58%). In 2 of them, length-dependent polyneuropathy was detected by routine NCSs. Both reduced SNAP of DSN and abnormal sural/DSN SNAP ratio were evident in further 10 individuals with normal findings on standard NCSs (Table 2 and Supplementary Table 1).

Twenty-one/38 carriers (55.26%) showed abnormal findings in at least one test aimed at exploring small nerve fibers (including both routine and “unconventional” exams). Globally, Sudoscan revealed abnormal findings in 13 cases (also including those cases with mild/borderline values), skin biopsy in 11 cases, and CCM in all the 7 examined cases, whereas CSP was absent in 2 cases (Table 2 and Supplementary Table 1). Interestingly, observed findings were suggestive of a non-length-dependent pattern in 11 cases (i.e., 9 cases documented by skin biopsy, 2 cases by Sudoscan).

### sNfL

Serum levels of NfL were available for 35 out of 38 carriers (all the 16 “converted” carriers *vs.* 19 out of 22 still-presymptomatic carriers) (Supplementary Table 1).

Values above the cut-off threshold of 37.10 pg/mL were detected only in the subgroup of “converted” carriers (9/16).

Mean values of sNfL were significantly higher in this subgroup (44.91 ± 39.34 pg/mL, median 37.14, IQR 11.42–74.78) than in still-presymptomatic carriers (13.67 ± 10.56 pg/mL, median 11.70 pg/ml, IQR 4.12–21.20; mean difference 31.24 pg/mL; *p* = 0.005).

### Comparisons between the whole subgroup of “converted carriers” and the subgroup of still-presymptomatic carriers

Considering the whole subgroup of “converted” carriers (*n* = 16), these individuals were significantly older (61.19 ± 11.51 years) as compared to the subgroup of still-presymptomatic carriers (*n* = 22; 50.05 ± 10.22 years) (mean difference 11.14 years; *p* = 0.003). However, there was not any statistically significant difference between these 2 subgroups in terms of time-to-PADO, although being shorter in the first group (− 2.06 ± 12.77 years *vs.* − 7.59 ± 10.94 years; mean difference 5.53 years; *p* = 0.160).

## Discussion

In this study, we aimed to evaluate the potential use of supplementary, not yet validated investigations, in addition to standardized diagnostic tests, in the early diagnosis of symptomatic ATTRv-PN amyloidosis. In our cohort, it was possible to confirm disease onset using conventional instrumental tools in approximately 10% of individuals (4/38) during their periodic monitoring. Indeed, in further 12 cases (nearly 32%), one or more “unconventional” tests resulted abnormal, suggesting a possible “conversion” to overt disease also in those cases showing normal or not definitive findings on clinical examination and routine investigations. Interestingly, in many cases, conversion occurred long before PADO (up to 25 years earlier), supporting the importance of starting monitoring even before the 10-year cut-off.

Defining disease onset in ATTRv amyloidosis is challenging, as there is significant variability in disease expression across individuals and geographic areas. Moreover, ATTRv amyloidosis is a multisystemic disease, and detecting the first involved tissue requires a multidisciplinary approach carried out by expert specialists from across many fields.

From the neurological point of view, in the follow-up of *TTR* mutation carriers, an extensive neurological examination and traditional NCSs are essential to identify and evaluate the extent of peripheral nerve involvement. NCSs are also essential as longitudinal assessment measures to detect any change over time from the baseline in neurophysiological data [6, 38]. Such a change may indeed precede by as much as 2 years the onset of symptoms [38].

The sural nerve is the sensory nerve most commonly assessed in the diagnostic workup of suspected length-dependent polyneuropathy. However, the segment routinely explored in daily practice is proximal to the sites initially affected by distal polyneuropathies. The *study of the sural lateral dorsal cutaneous branch* could overcome this limitation, therefore allowing early detection of peripheral nerve damage [27, 31, 39]. In our cohort, longitudinal conventional NCSs were able to detect sensory polyneuropathy as the first disease sign only in 2 cases. Conversely, the study of the DSN proved abnormal in more than 30% of the examined carriers, supporting its potential role in early diagnosis of ATTRv-PN amyloidosis.

Moreover, routine electrophysiological studies are often completely normal in those cases characterized by early, exclusive involvement of small nerve fibers (SFs). Importantly, SF involvement is often the first manifestation in early-onset ATTRv amyloidosis, while it is usually considered less prominent in late-onset cases, especially in the very early stages.

Several non-invasive techniques, such as sympathetic skin response (SSR), QST, and laser evoked potentials (LEPs), can be useful to investigate small-fiber neuropathy (SFN) [40, 41]. However, all of them are time-consuming and not available anywhere.

*Sudoscan* is a simple, quick diagnostic test, whose role in ATTRv amyloidosis has already been assessed [42–44], showing a high diagnostic accuracy, especially in the early-onset population. Hence, its application as a screening test in *TTR* mutation carriers has already been recommended by the 2018 Consensus, mainly in endemic areas. Conversely, in non-endemic countries (such as Italy), although a demonstrated role in monitoring disease progression and severity [43, 44], its utility for early diagnosis has always been considered uncertain. However, in our cohort, 2 people first developed confirmed SFN (documented by Sudoscan), with initial sparing of large-diameter myelinated nerve fibers. Slightly abnormal ESC values have been documented in further 11 individuals. All but 4 (7/11) presented at least another abnormal test, confirming the value of this tool in the early detection of small nerve fiber impairment also in the presence of mild/borderline sudomotor dysfunction.

The *cutaneous silent period* and in vivo* corneal confocal microscopy* are further non-invasive, easy-to-perform techniques aimed at exploring SFs. Even if underused, they seem to have an emerging role in the monitoring of disease onset and progression in ATTRv amyloidosis.

CSP is a spinal inhibitory reflex whose afferent arc is mediated almost exclusively by high-threshold, Aδ sensory nerve fibers [32, 45–47]. Despite the absence of normative data, recent reports suggest an early alteration of CSP in terms of onset latency and duration both in ATTRv patients and presymptomatic carriers [37, 48].

CCM*,* providing in vivo imaging of corneal nerve fibers, represents another promising sensitive tool in the early detection of nerve damage in peripheral neuropathies mainly affecting small fibers [49], including ATTRv-PN amyloidosis [21, 50, 51]. In patients with SFN, abnormalities on CCM may sometimes even precede loss of intraepidermal nerve fibers in skin biopsies [49], as supported by our findings as well.

Nevertheless, although being available only in a few highly specialized centers, *skin biopsy* still represents the gold standard for the diagnosis of SFN. Providing information about somatic and autonomic SFs, this minimally invasive technique appears to be a promising tool in the assessment of presymptomatic *TTR* mutation carriers. Interestingly, skin biopsy can disclose pathological changes even several years before the onset of symptoms, supporting the role of IENFD quantification as a possible biomarker for subclinical ATTRv disease [7]. Moreover, this technique can help to shed light on pathophysiologic mechanisms underlying SFNs. The leg/thigh IENFD ratio can be used indeed as a parameter to discriminate between length-dependent small-fiber neuropathy and small-fiber sensory ganglionopathy [34]. Curiously, in a recent paper by Leonardi et al., skin denervation was sometimes more pronounced in proximal than distal sites, suggesting a non-length-dependent pattern in at least a subgroup of subjects [7]. In line with literature data, a reduced IEFND was evident in more than half of the examined cases of our cohort, with most cases (9/11) showing findings suggestive of a non-length-dependent involvement.

Globally, more than half of our study cohort (21/38) showed abnormal findings on one or more (either invasive or non-invasive) tests aimed at exploring SFs. Altogether, our data support the importance of exploring not only large nerve fibers but also small nerve fibers in the monitoring of *TTR* presymptomatic carriers, independently from the geographic origin.

Interestingly, in a subgroup of individuals, observed findings were suggestive of a non-length-dependent pattern. As a matter of fact, even if progressive, length-dependent polyneuropathy is the most common neurological presentation in non-endemic areas, other phenotypes, including multifocal neuropathy with onset in the upper limbs, have been reported as well [52].

## Conclusions

Overall, our data support the importance of the periodic monitoring of *TTR* mutation carriers, suggesting the need for broadening the neurological diagnostic workup in the context of a multidisciplinary evaluation. This is fundamental to detect disease onset at the earliest convenience, since a subclinical stage, to effectively modify the disease’s natural history and prognosis.

However, we cannot certainly imagine the actual application of all these techniques in daily clinical practice. It should be thus necessary to define the most accurate tests in terms of sensibility and specificity to avoid performing unnecessary “overlapping” investigations, aimed at exploring the same system. In addition to the investigations already approved and recommended by the Consensus, from a neurological point of view, it would be worth applying the study of the dorsal sural nerve and the skin biopsy (or the corneal confocal microscopy) in order to combine a neurophysiological test aimed at exploring the large nerve fibers (probably with a higher sensibility as compared to standard NCSs) with a technique intended to assess small nerve fiber involvement.

Unfortunately, in our study, although having an adequate cohort of subjects for such a rare disease, the various mentioned methods were not employed in all individuals. Therefore, we cannot draw any conclusions in terms of the most reliable, sensitive tests for early diagnosis of symptomatic disease.

Long-term follow-up of carriers and further longitudinal studies on large and diversified cohorts are needed to define the best combination of tests to be employed to reach the highest diagnostic accuracy.

### Supplementary Information

Below is the link to the electronic supplementary material.

Supplementary file1 (DOCX 31.6 KB)

## Data Availability

The data that support the findings of this study are available from the corresponding author upon reasonable request.
