# Supplementary material for: Early detection of nerve involvement in presymptomatic TTR mutation carriers: exploring potential markers of disease onset
Source: Neurol Sci. 2023 Nov 8;45(4):1675–84. doi: 10.1007/s10072-023-07177-x (PMC10942905; doi:10.1007/s10072-023-07177-x)
Supplement: Supplementary file 1 — Supplementary file1 (DOCX 31.6 KB) [file 10072_2023_7177_MOESM1_ESM.docx]

| **ID** | **Age at conversion or last FU** | **PADO** | **time-to-PADO** (years) | **Symptoms *possibly* related to ATTRv** | **Routine tests** | | **"Unconventional" tests** | | | | | **sNfL**  (pg/mL) |
| --- | --- | --- | --- | --- | --- | --- | --- | --- | --- | --- | --- | --- |
|  |  |  |  |  | **Conventional  NCS**  (R/L sural SNAP, μV) | **Sudoscan**  (LL/UL ESC, μS) | **NCS of DSN**  (R/L DSN SNAP, μV) | **Sural/DSN SNAP ratio**  (R/L) | **CSP**  (present/  absent) | **Skin biopsy**  (distal IENFD,  fibers/mm) | **CCM** |  |
| **F#1*** | 68 | 55 | 13 | Y | 10.8/10.0 | *64*/57 | *7.4*/*5.9* | 1.46/1.69 | present | *3.90* | N/A | 18.40 |
| **M#2*** | 63 | 55 | 8 | N | 12.0/9.0 | *67*/*59* | *2.7*/*3.0* | *4.44*/3.00 | present | *7.40* | N/A | *75.70* |
| F#3 | 45 | 56 | -11 | N | 13.3/11.7 | *65*/65 | *3.8*/*4.2* | 3.50/2.79 | present | 15.30 | N/A | 3.66 |
| M#4 | 42 | 56 | -14 | N | 10.5/12.8 | *53*/*56* | *7.9*/*6.0* | 1.33/2.13 | present | 16.60 | N/A | 24.70 |
| M#5 | 60 | 60 | 0 | Y | 11.1/12.0 | 73/64 | *2.9*/*3.6* | 3.83/3.33 | present | 10.60 | N/A | 17.90 |
| F#6 | 55 | 72 | -17 | N | 31.4/38.9 | 71/83 | *4.2*/*7.6* | *7.48*/*5.12* | present | N/A | N/A | 8.61 |
| **F#7*** | 59 | 63 | -4 | Y | 17.9/20.4 | 78/78 | *2.4*/*7.2* | *7.46***/**2.83 | present | *7.50* | abnormal | 9.29 |
| M#8 | 64 | 63 | 1 | N | 10.4/10.2 | *66*/81 | *3.8*/7.2 | 2.74/1.42 | present | N/A | N/A | 13.30 |
| M#9 | 69 | 58 | 11 | N | 10.3/9.9 | 80/70 | *4.5*/*4.8* | 2.29/2.06 | present | N/A | N/A | 33.50 |
| M#10 | 49 | 51 | -2 | N | 20.9/22.1 | 81/82 | 8.8/9.7 | 2.38/2.28 | present | *7.12* | N/A | 11.40 |
| **F#11*** | 43 | 51 | -8 | Y | 22.0/26.6 | 74/75 | *4.2*/*5.4* | *5.24*/*4.93* | present | 14.22 | abnormal | 6.50 |
| M#12 | 49 | 75 | -26 | N | 25.3/22.4 | 72/72 | *5.7*/*6.2* | *4.44*/3.61 | present | N/A | N/A | N/A |
| **F#13** | 58 | 76 | -18 | N | *7.4*/*7.5* | 86/77 | N/A | N/A | N/A | N/A | N/A | 18.20 |
| M#14 | 53 | 51 | 2 | N | 25.0/22.6 | 82/76 | *4.7*/7.4 | *5.32*/3.05 | present | N/A | N/A | 21.20 |
| **M#15** | 51 | 76 | -25 | N | *8.1*/*7.3* | 80/76 | N/A | N/A | N/A | N/A | N/A | *140.00* |
| **M#16*** | 42 | 63 | -21 | N | 34.8/30.3 | 80/76 | *7.3*/10.5 | *4.77*/2.89 | present | *8.92* | N/A | 7.57 |
| F#17 | 38 | 63 | -25 | N | 17.5/19.5 | 71/71 | *5.2*/*7.1* | 3.37/2.75 | present | 11.85 | abnormal | N/A |
| M#18 | 50 | 63 | -13 | N | 16.8/15.0 | 76/73 | *5.9*/*5.1* | 2.85/2.94 | present | 14.99 | abnormal | 30.70 |
| M#19 | 51 | 63 | -12 | N | 20.5/17.3 | 83/79 | 8.9/*5.4* | 2.30/3.20 | present | N/A | N/A | 4.12 |
| **M#20** | 54 | 60 | -6 | N | 14.9/15.2 | *46*/*48* | N/A | N/A | N/A | *7.0* | N/A | *103.00* |
| F#21 | 43 | 35 | 8 | Y | 16.3/13.5 | 79/73 | N/A | N/A | N/A | N/A | N/A | N/A |
| **F#22*** | 73 | 61 | 12 | Y | 12.6/16.6 | *65***/***51* | *3.7*/*2.7* | 3.41/*6.15* | present | *9.58* | abnormal | *37.15* |
| **F#23*** | 75 | 61 | 14 | Y | 14.8/17.0 | *57***/**69 | N/A | N/A | N/A | *8.33* | N/A | *76.30* |
| **F#24*** | 71 | 63 | 8 | Y | 13.1/11.2 | *53***/***48* | N/A | N/A | N/A | *6.37* | N/A | *43.50* |
| **F#25*** | 74 | 70 | 4 | N | 14.7/12.6 | *60*/77 | N/A | N/A | N/A | *6.70* | abnormal | *37.12* |
| **M#26*** | 72 | 67 | 5 | Y | 10.1/11.1 | 76/*57* | *2.2*/*2.2* | *4.59*/*5.05* | present | N/A | N/A | *72.00* |
| M#27 | 68 | 67 | 1 | N | 10.2/9.3 | 73/*52* | *2.6*/*2.5* | 3.92/3.72 | present | N/A | N/A | 33.70 |
| **F#28** | 71 | 63 | 8 | Y | 14.8/18.1 | *45*/*33* | N/A | N/A | N/A | N/A | N/A | *52.00* |
| F#29 | 42 | 50 | -8 | N | 15.2/15.0 | 73/69 | *8.8*/*8.7* | 1.73/1.72 | present | N/A | N/A | 11.70 |
| M#30 | 40 | 54 | -14 | N | 36.7/43.3 | 78/70 | 19.7/35.1 | 1.86/1.23 | present | N/A | N/A | 1.82 |
| F#31 | 43 | 54 | -11 | N | 29.8/26.7 | 78/69 | 19.9/15.3 | 1.50/1.75 | present | 12.45 | N/A | 5.80 |
| F#32 | 68 | 54 | 14 | Y | 10.3/9.9 | 81/85 | *4.3*/*4.2* | 2.40/2.36 | present | N/A | N/A | 12.50 |
| F#33 | 39 | 54 | -15 | N | 31.0/29.9 | 85/77 | *11.2*/*11.5* | 2.77/2.60 | present | 16.26 | N/A | 1.86 |
| **M#34*** | 45 | 56 | -11 | Y | 16.0/19.5 | 84/87 | *7.1*/12.4 | 2.25/1.57 | absent | N/A | abnormal | 4.05 |
| **M#35*** | 60 | 72 | -12 | Y | 9.9/17.1 | 72/72 | *4.7*/*3.1* | 2.11/*5.52* | absent | *5.70* | N/A | 17.80 |
| M#36 | 35 | 54 | -19 | N | 18.4/19.0 | 86/80 | *6.6*/*6.8* | 2.79/2.79 | present | N/A | N/A | 2.10 |
| F#37 | 47 | 50 | -3 | Y | 31.8/24.0 | 85/86 | 11.8/11.9 | 2.69/2.02 | present | N/A | N/A | 12.40 |
| M#38 | 51 | 65 | -14 | N | 12.4/12.2 | 80/74 | *4.4*/*4.3* | 2.82/2.84 | present | N/A | N/A | 8.82 |

**Supplementary table 1.** The main findings on routine and “unconventional” tests in the whole study cohort at the time of “conversion” or last follow-up, depending on the individual case. Normal tests are marked in “green”, abnormal ones in “red”; abnormal values are written in italics. As concerns the Sudoscan, the “red” cells refer to *definitely* abnormal values (suggestive of severe sudomotor dysfunction), the “yellow” ones to low/borderline values (indicative of mild/borderline sudomotor dysfunction), whereas the “green” cells correspond to normal values, according to the manufacturer's reference intervals [30]. For the sural and dorsal sural nerve SNAPs, age- and sex-adjusted normative values of our laboratory were used [28, 29, 31]. The “converted” carriers (defined according to Consensus’ minimum criteria [6]) are marked in bold. The asterisk denotes the subgroup of carriers which were considered “converted” based on "unconventional" tests or on combination of both routine and “unconventional” tests. *Abbreviations*: R, right; L, left; SNAP, sensory nerve action potential; ESC, electrochemical skin conductance; DSN, dorsal sural nerve; CSP, cutaneous silent period; IENFD, intraepidermal nerve fiber density; CCM, *in-vivo* corneal confocal microscopy; sNfL, serum levels of neurofilament light chain; M, male; F, female; Y, yes; N, no; N/A, not available.
